# Variability in cTBS Aftereffects Attributed to the Interaction of Stimulus Intensity With BDNF Val66Met Polymorphism

**DOI:** 10.3389/fnhum.2021.585533

**Published:** 2021-06-18

**Authors:** Denise Y. Harvey, Laura DeLoretta, Priyanka P. Shah-Basak, Rachel Wurzman, Daniela Sacchetti, Ahmed Ahmed, Abdou Thiam, Falk W. Lohoff, Olufunsho Faseyitan, Roy H. Hamilton

**Affiliations:** ^1^Department of Neurology, University of Pennsylvania, Philadelphia, PA, United States; ^2^Research Department, Moss Rehabilitation Research Institute, Philadelphia, PA, United States; ^3^National Institute on Alcohol Abuse and Alcoholism, National Institutes of Health (NIH), Bethesda, MD, United States

**Keywords:** brain-derived neurotropic factor (BDNF) gene, motor plasticity, neurorehabilitation, motor-evoked potentials, continuous theta burst stimulation (cTBS)

## Abstract

**Objective**: To evaluate whether a common polymorphism (Val66Met) in the gene for brain-derived neurotrophic factor (BDNF)—a gene thought to influence plasticity—contributes to inter-individual variability in responses to continuous theta-burst stimulation (cTBS), and explore whether variability in stimulation-induced plasticity among Val66Met carriers relates to differences in stimulation intensity (SI) used to probe plasticity.

**Methods**: Motor evoked potentials (MEPs) were collected from 33 healthy individuals (11 Val66Met) prior to cTBS (baseline) and in 10 min intervals immediately following cTBS for a total of 30 min post-cTBS (0 min post-cTBS, 10 min post-cTBS, 20 min post cTBS, and 30 min post-cTBS) of the left primary motor cortex. Analyses assessed changes in cortical excitability as a function of BDNF (Val66Val vs. Val66Met) and SI.

**Results**: For both BDNF groups, MEP-suppression from baseline to post-cTBS time points decreased as a function of increasing SI. However, the effect of SI on MEPs was more pronounced for Val66Met vs. Val66Val carriers, whereby individuals probed with higher vs. lower SIs resulted in paradoxical cTBS aftereffects (MEP-facilitation), which persisted at least 30 min post-cTBS administration.

**Conclusions**: cTBS aftereffects among BDNF Met allele carriers are more variable depending on the SI used to probe cortical excitability when compared to homozygous Val allele carriers, which could, to some extent, account for the inconsistency of previously reported cTBS effects.

**Significance**: These data provide insight into the sources of cTBS response variability, which can inform how best to stratify and optimize its use in investigational and clinical contexts.

## Introduction

Transcranial magnetic stimulation (TMS) has received considerable attention in both research and clinical settings due to its ability to probe and transiently modulate cortical activity. In clinical contexts, TMS is routinely employed as a treatment for depression (Connolly et al., 2012), and recently as a treatment for migraine and obsessive-compulsive disorder (Schwedt and Vargas, 2015; Voelker, 2018) and numerous other neurologic and psychiatric conditions (Schlaepfer et al., 2010). In research settings, it has proven to be a powerful tool for interrogating brain structure-function relationships, pertaining to a wide range of motor and cognitive abilities (Devlin and Watkins, 2006; Lowe et al., 2018; Medaglia et al., 2018) as well as social (Ferrari et al., 2016; Era et al., 2018, 2020) and emotional processes (Moors et al., 2019; Fini et al., 2020), and to characterize and index fundamental neurophysiologic properties, including but not limited to cortical excitability (Pascual-Leone et al., 1998), interhemispheric interactions (Mochizuki et al., 2004), and activity-induced neuroplasticity (Bolognini et al., 2009). However, the findings from these studies are often muddled by the amount of inter- and intra-individual variability that is typically observed in response to stimulation (Ridding and Ziemann, 2010; Chung et al., 2016). Identifying and controlling for factors that contribute to this variability, both in the physiologic and behavioral aftereffects, is an important strategy for optimizing the investigative and therapeutic utility of TMS protocols.

In recent years, there is a growing interest in theta-burst stimulation (TBS), which is a modified form of repetitive TMS (rTMS). TBS reportedly interferes with cortical excitability and produces aftereffects to the same degree as the conventional rTMS protocols but in a fraction of the time; the application of TBS can take 20–190 s compared to 15–30 min of conventional rTMS protocols. While the short implementation time has made TBS a very attractive tool for research and clinical investigations, it is not immune to the observed response variability.

A TBS pattern consists of 50 Hz bursts of stimulation pulses delivered in triplets every 200 ms (at 5 Hz). Depending on the number of repetitions of the TBS pattern and the interval between repetitions, TBS can increase or decrease cortical excitability. Across studies, TBS has typically been found to be excitatory with intermittent delivery of TBS pattern (iTBS) and inhibitory with continuous TBS (cTBS) pattern (Huang et al., 2005). While the induced changes in cortical excitability with iTBS and cTBS are found at the group level, further evaluations of the data indicate that there is considerable variability in response to TBS, both within and between individuals (Ridding and Ziemann, 2010; Goetz et al., 2014; Hordacre et al., 2017; Corp et al., 2020). Evidence indicates that extrinsic factors related to stimulation parameters and experimental design such as the degree of pre-activation of targeted muscles (Iezzi et al., 2008; Goldsworthy et al., 2014), the stimulation intensity (SI) of single-pulse TMS for probing cortical excitability with TBS (Vallence et al., 2015; Goldsworthy et al., 2016b), and the SI of TBS pulse triplets (Jannati et al., 2017; Sasaki et al., 2018) contribute to this variability. In addition, properties that are intrinsic to each individual such as age (Freitas et al., 2011) and genetic factors can also contribute to the variability.

One of the most notable genetic factors that has been purported to affect learning, memory, and neuroplasticity is the brain-derived neurotrophic factor (BDNF; e.g., Cheeran et al., 2008; Jannati et al., 2017). BDNF is a protein encoded by the BDNF gene, supporting the survival, growth, and differentiation of neurons and synapses (Huang and Reichardt, 2001). BDNF plays an important role in synaptic plasticity as its release is thought to aid long-term potentiation (LTP) and long–term depression (LTD) processes (Lu, 2003). A relatively common polymorphism in the BDNF gene (Val66Met allele) is associated with a decrease in activity-dependent release of BDNF (McHughen et al., 2009) and diminished synaptic plasticity in animal models (Ninan et al., 2010). Evidence suggests that this polymorphism is also associated with impairments in learning and memory in humans (Bath and Lee, 2006; Soliman et al., 2010). Given that the persistent effects of rTMS—including TBS—are believed to be mediated by LTP- or LTD-like effects on synaptic plasticity, individuals with the Val66Met allele may be less responsive to TBS. However, evidence to support this hypothesis has been mixed. While some studies have found that Val66Met allele carriers exhibit little-to-no aftereffects of TBS when compared to their homozygous (Val66Val) counterparts (Cheeran et al., 2008; Antal et al., 2010; Lee et al., 2013; Jannati et al., 2017), others report no difference in susceptibility to TBS as a function of BDNF genotype status (Li Voti et al., 2011; Mastroeni et al., 2013). One possible explanation for this discordance between studies is that Val66Met allele carriers may be more variable than Val66Val homozygotes with respect to response to brain stimulation. A recent meta-analysis supports this view: Chung et al. (2016) found that Val66Val carriers show more consistent response across studies, with greater effect sizes for iTBS. By contrast, Val66Met carriers were more variable (also see Jannati et al., 2019).

Here, we ask whether increased variability in cTBS response among Val66Met allele carriers relates to the SI of single-pulse TMS used to probe cortical excitability before and immediately after cTBS; we refer to the single-pulses as test pulses in this study. Prior work has shown that the SI of test pulses impacts cTBS-induced suppression of motor evoked potentials (MEPs; Vallence et al., 2015; Goldsworthy et al., 2016b). This is directly relevant to studies involving TBS because different approaches have been employed by different investigators to establish SI. For example, some studies determine SI based on the resting motor threshold (rMT), while others stimulate at an SI that is empirically determined to be sufficient to generate an MEP of a certain amplitude, often 1 mV (SI_1mV_). These methodological differences in SI determination may introduce noise, especially if SI differentially affects cTBS response in Val66Val vs. Val66Met carriers (Cheeran et al., 2008; Antal et al., 2010; Lee et al., 2013; Jannati et al., 2017). In the current study, we re-examine the impact of BDNF genotype status on cTBS-induced suppression of motor excitability. We hypothesized that cTBS aftereffects for BDNF Met allele carriers would be less reliable, and investigated whether differences in SI [as determined using the percentage of maximum stimulator output (MSO) required to produce MEPs with a peak-to-peak amplitude of ~1 mV] account for variable cTBS responses as a function of BDNF genotype status.

## Materials and Methods

### Overview

**Figure 1:**
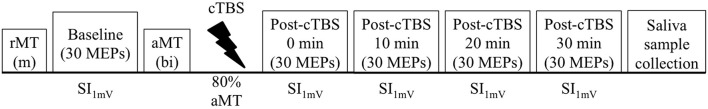
Overview of experimental design. Thirty motor evoked potentials (MEPs)were collected before (Baseline), immediately after continuous theta-burst stimulation (cTBS) administration (Post-cTBS 0 min), and in 10 min intervals up to 30 min after administration of cTBS(Post-cTBS 10 min, Post-cTBS 20 min, and Post-cTBS 30 min). Stimulation intensity (SI) was individually adjusted to produce MEPs of approximately 1 mV (SI_1mV_), as determined prior to cTBS. Resting motor threshold (RMT) was determined using a monophasic (m) pulse waveform, whereas active motor threshold (aMT) was determined using a biphasic (bi) pulse waveform.

The experiment consisted of a single session. Participants were seated in a comfortable chair with their right arm resting on a pillow and were instructed to keep their right hand relaxed throughout the duration of the experiment. Single TMS pulses were delivered over the left primary motor cortex. We first determined the rMT, and then gradually increased SI to the percentage MSO required to produce MEPs with peak-to-peak amplitudes of approximately 1 mV (SI_1mV_). Prior to cTBS (Baseline) and in 10 min intervals immediately following cTBS for a total of 30 min post-cTBS (0 min post-cTBS, 10 min post-cTBS, 20 min post cTBS, and 30 min post-cTBS), we collected 30 MEPs from the first dorsal interosseous (FDI) of the dominant (right) hand at SI_1mV_, following the recommendation from prior research (Goldsworthy et al., 2016a). Test pulses were delivered with an inter-stimulus interval of 6 s with a random jitter of ±6%. Approximately 10–15 min following baseline MEP collection and 10–15 min prior to cTBS administration, we obtained active motor threshold (aMT). Following post-cTBS MEP collection, we obtained saliva samples for BDNF genotyping (Figure 1).

### Participants

Thirty-three neurologically healthy individuals (16 females) aged 18–45 [mean (M) ± standard deviation (SD) = 24.6 ± 6.2 years] with no contraindications to TMS participated in the study. All participants provided informed consent in accordance with the Institutional Review Board at the University of Pennsylvania. See Supplementary Table 1 for demographic and stimulation parameter data for each participant.

### Electromyography

Electromyographic (EMG) activity was recorded using surface electrodes overlying the belly of the right FDI. Signals were amplified and band-pass-filtered between 20 and 2,000 Hz, digitized **(**sample-rate 5 kHz), and stored for offline analysis using SIGNAL software (Cambridge Electronic Devices, Cambridge, UK).

### Transcranial Magnetic Stimulation

Single-pulse TMS with monophasic waveform was administered using a hand-held figure-of-eight coil (Magstim Company, Whitland, Dyfield, UK). The coil was positioned over the left motor cortex (M1) to a site that reliably elicited an MEP in the right FDI muscle (i.e., the motor hotspot). The Brainsight (Rogue Inc., Montreal, QC, Canada) neuronavigational system was used to mark the motor hotspot on native-space magnetic resonance image volumes collected prior to the experiment. In line with widely accepted methods, rMT was defined as the minimum pulse intensity required to elicit MEPs with peak-to-peak amplitudes of at least 50 μV in 5 of 10 consecutive trials with the FDI at rest (Rossini et al., 1999; Rothwell et al., 1999; Schlaepfer et al., 2010).

### Continuous Theta-Burst Stimulation

CTBS was administered with a biphasic waveform using a Magstim SuperRapid^2^ Stimulator (Whitland, UK). Following the standard procedure (Huang et al., 2005), cTBS entailed continuous delivery of 50 Hz triplets of TMS pulses at 5 Hz for a total of 600 pulses (~40 s). SI of cTBS pulses was set to 80% of aMT, defined as the minimum pulse intensity required to produce MEPs with peak-to-peak amplitudes of at least 200 μV in 5 of 10 consecutive pulses while participants contracted the right FDI muscle at approximately 20% of the maximal voluntary contraction. EMG activity was displayed to participants in real-time using SIGNAL software (Cambridge Electronic Devices, Cambridge, UK) in order to ensure they maintained approximately 20% of maximal voluntary contraction of the right FDI immediately prior to and during the delivery of single-pulse TMS. Participants were instructed to relax the right FDI in between test pulses. The same biphasic stimulator was used to determine aMT and administer cTBS.

### BDNF Genotyping

Genomic DNA from human saliva samples was collected in Oragene^®^ DNA collection kits and was then isolated using the prepIT.L2Preagent (cat # PT-L2P-5, DNA Genotek Inc., Canada) and precipitated with ethanol according to manufacturer’s instructions. The DNA samples were genotyped for BDNF (the single nucleotide polymorphism rs6265) using the TaqMan SNP Genotyping Assay (C__11592758_10) designed by Thermo Fisher Scientific. Primers and probes were mixed with TaqMan^®^ Universal PCR Master Mix (Thermo Fisher Scientific). 4.5 μl of genomic DNA (2.5 ng/μl) was transferred in triplicate to a 384-well plate, with each well containing 5.5 μl of the PCR mixture. The PCR reaction was performed following a protocol provided by ABI. The allele was discriminated by post-PCR plate reading on the ViiA™ 7 System. Data were processed using the ViiA™ 7 Software (Thermo Fisher Scientific).

### Statistical Analyses

We excluded from analyses MEPs with amplitudes greater than two SDs from a participant’s mean MEP amplitude within each timepoint (baseline vs. 0, 10, 20, and 30 min post-cTBS), resulting in the removal of 4.6% of all trials (baseline = 4.4%; 0 min post-cTBS = 4.7%; 10 min post-cTBS = 5.0%; 20 min post-cTBS = 4.2%; 30 min post-cTBS = 4.5%)^1^. MEPs at 30 min post-cTBS were not collected from three participants due to time constraints during the experiment session. Analyses were conducted on 4, 336 MEPs (baseline = 869; 0 min post cTBS = 865; 10 min post-cTBS = 877; 20 min post-cTBS = 902; 30 min post-cTBS = 823) using linear mixed effects modeling (Baayen et al., 2008)—an analysis approach that has been utilized in prior neurophysiological studies of stimulation-induced plasticity (see e.g., Goetz et al., 2016; Moret et al., 2019)—implemented in the lme4 package (Bates et al., 2015) of R version 3.6.3 (R Core Team, 2020). We adopted a model comparison approach to determine whether the factors under investigation impact cTBS-induced changes in motor excitability above and beyond contributions from other factors that may affect MEP amplitudes and cTBS responsiveness. To account for age-related changes in neuroplasticity (Freitas et al., 2011), we first fit a base model with age as a covariate and sequentially added to the base model the fixed effect timepoint (baseline vs. 0, 10, 20, and 30 min post-cTBS). We then sequentially added the remaining fixed effects of interest (the categorical predictor representing BDNF status [Val66Val vs. Val66Met] and the continuous predictor representing the range of SI_1mV_ values) and tested whether the inclusion of each factor and/or interaction term significantly improved the model fit using the change in the deviance statistic (−2 times the log-likelihood), which follows a chi-square distribution with degrees of freedom equal to the number of parameters added. The main advantage of this analytic approach is that it improves the interpretability and generalizability of findings by accounting for the random effects that can influence model parameter estimates, resulting in more precise estimates of the fixed effects of interest (e.g., Warrington et al., 2014; Harrison et al., 2018). Moreover, it is robust to violations of normality (e.g., Zuur et al., 2010) and unequal group sizes (here, unequal n per BDNF genotype group) when compared to regression analyses conducted on mean values per subject (e.g., Field and Wright, 2011). Nonetheless, graphical tools for assessing linear mixed model assumption violations [e.g., normal quantile-quantile (QQ) plot of residuals] were evaluated (following the guidelines of Zuur et al., 2010; see also Harrison et al., 2018), revealing that the assumptions had not been violated.

All models included by-participant random intercepts to capture the inherent correlation among multiple measurements within a participant and adjust for individual differences present prior to the intervention. Following the recommendation of Barr et al. (2013), we attempted to implement a maximal random effects structure reflecting by-BDNF genotype status random slopes for the fixed effect of timepoint, as other fixed effects of interest were not fully crossed within participants (i.e., SI and BDNF genotype status) and the inclusion of by-participant random slopes of timepoint are not warranted theoretically given our prediction that the slope of the timepoint effect differs as a function of BDNF genotype status. However, this maximal random effects structure triggered convergence warnings (indicative of model overfitting), and did not significantly improve model fit (*p* = 1).

We conducted *post hoc* comparisons using the emtrends function in the Estimated Marginal Means R package (Lenth, 2020). We first assessed whether the change in MEPs from baseline to each post-cTBS timepoint differed as a function of the slope of SI within each BDNF genotype group separately, which allowed us to evaluate whether MEPs significantly change from baseline within each group and if that change differs as a function of increasing SI. We then evaluated between-group differences in MEPs across the slope of timepoint for the median of the upper and lower tercile of SI values (SI_65_ and SI_44_, respectively) in order to assess whether the two groups differ in terms of how higher/lower SI affects change in MEPs from baseline to post-cTBS timepoints. The Tukey method was used to correct for multiple comparisons within each family of estimates.

## Results

Among the 33 participants, 20 were Val66Val carriers, 11 were Val66Met carriers and two were Met66Met carriers, consistent with the known prevalence of BDNF Met allele polymorphism (Shimizu et al., 2004). However, because there were only two homozygous Met allele carriers in the current sample and the extent to which Met66Met carriers behave similarly to heterozygous Met allele carriers remains to be clarified (e.g., Egan et al., 2003), data from these two participants were excluded from analyses (rather than collapsed into the Val66Met group; see e.g., Cheeran et al., 2008). Comparisons between the two groups revealed that there were no significant differences in age [Val66Val mean (*M*) ± standard deviation (*SD*) = 23.5 ± 5.7 vs. Val66Met *M* = 25.5 ± 7.0] or mean MEP amplitudes at baseline (Val66Val *M* = 1.03 ± 0.2 mV vs. Val66Met *M* = 0.86 ± 0.4 mV; *p*’s > 0.10). Although rMT significantly differed for Val66Val (*M* = 48.7 ± 8.3) vs. Val66Met carriers (*M* = 41.6 ± 8.1; *p* = 0.03), there were no significant differences in SI—whether defined relative to the individual (% rMT; Val66Val *M* = 116.3 ± 7.4 vs. Val66Met *M* = 120.2 ± 14.2) or the stimulator (MSO; Val66Val *M* = 56.5 ± 9.8 vs. Val66Met *M* = 49.7 ± 9.4; *p*’s > 0.07). Thus, the two groups did not differ with respect to SI_1mV_ used to collect MEPs (Table 1). All subjects tolerated the cTBS with no adverse effects.

**Table 1:** Results of the independent-samples *t*-tests comparing demographic and stimulation parameters for BDNF Val66Val vs. Val66Met allele carriers.

	*Test*	*Statistic*	*df*	*p*	Effect Size	Val66Val	Val66Met
						*M ± SD*	*M ± SD*
*N*						20	11
Age	*U*	118.50		0.74	0.08	23.5 ± 5.7	25.5 ± 7.0
rMT	*t*	2.27	29	0.03	0.85	48.7 ± 8.3	41.6 ± 8.1
SI_1mV_	*t*	1.87	29	0.07	0.70	56.5 ± 9.8	49.7 ± 9.4
% rMT	*U*	117.00		0.79	0.06	116.3 ± 7.4%	120.2 ± 14.2%
MEP_pre-cTBS_	*U*	69.00		0.10	0.37	1.03 ± 0.2 mV	0.86 ± 0.4 mV

*Note. Independent samples *t*-tests were performed unless data violated the assumption of normality and/or equal variances, in which the Mann-Whitney (*U*) tests were performed. Effect sizes for *t*-tests represent Cohen’s *d*, whereas effect sizes for Mann-Whitney tests represent the rank biserial correlation. SI_1mV_ (defined as the percentage of maximum stimulator output; MSO) and % rMT reflects the SI used to probe plasticity prior to and immediately following cTBS. BDNF, brain-derived neurotropic factor; MEP, motor evoked potential; rMT, resting motor threshold; SI, stimulation intensity; *M*, mean; *SD*, standard deviation; mV, millivolt*.

First, we assessed whether cTBS decreased motor excitability while accounting for potential age-related differences in cTBS response by adding to the base model the fixed effect of timepoint. Including the timepoint significantly improved model fit ($\chi_{\left(1\right)}^{2}$ = 27.97, *p* < 0.0001). We then evaluated the impact of BDNF genotype status on cTBS response by testing improvements in model fit (over that which included age and timepoint as single terms) with the addition of BDNF genotype status and the timepoint × BDNF interaction. Neither BDNF genotype status (*p* > 0.61) nor the interaction between BDNF genotype status and timepoint improved model fit (*p* > 0.45). We then explored whether test pulse SI (defined as MSO) impacted MEPs overall and/or cTBS-induced changes in cortical excitability as a function of BDNF genotype status. Adding SI did not improve model fit (*p* > 0.20), but including the interaction between BDNF, timepoint, and SI did significantly improve model fit ($\chi_{\left(6\right)}^{2}$ = 206.10, *p* < 0.0001). Model comparison results are reported in Table 2, and full model results including factor estimates and associated significance values are provided in Table 3.

**Table 2:** Model comparison results.

Model	logLik	dev	Chisq	df	*p*-value
MEP ~ Age + (1 | Subject)	−3987.5	7975.1			
MEP ~ **TimePoint** + Age + (1 | Subject)	−3973.5	7947.1	27.97	1	<0.0001
MEP ~ **BDNF** + TimePoint + Age + (1 | Subject)	−3973.4	7946.8	0.25	1	0.6169
MEP ~ BDNF*TimePoint + Age + (1 | Subject)	−3972.8	7945.5	1.57	2	0.4567
MEP ~ **SI_1mV_** + BDNF*TimePoint + Age + (1 | Subject)	−3971.3	7942.5	4.59	3	0.2043
MEP ~ SI_1mV_*BDNF*TimePoint + Age + (1 | Subject)	−3870.5	7741.0	206.10	6	<0.0001

*Note. The first row represents the base model, which includes covariates only. Subsequent rows illustrate the model comparison results after adding the fixed effect of interest highlighted in bold and comparing the model fit with that of the last significant model. The reference level for BDNF was set to Val66Val. Chisq, chi-squared; dev, deviance; df, degrees of freedom; TimePoint, slope of MEP change from baseline to 0, 10, 20, and 30 min post-cTBS; BDNF, brain-derived neurotropic factor (Val66Val vs. Val66Met); SI_1mV_, stimulation intensity [defined as percentage of maximum stimulator output (MSO)]; (1 | Subject), random effects structure representing the inclusion of a by-participant random intercept*.

**Table 3:** Mixed linear model coefficients and associated test statistics.

	*Coef*.	*SE*	*t*	*p*
Fixed effects				
Age	−0.029	0.015	−1.96	0.0498
**TimePoint**	**−0.227**	**0.048**	**−4.77**	**<0.0001**
BDNF	0.344	1.023	0.34	0.7365
SI_1mV_	0.003	0.011	0.27	0.7900
TimePoint*BDNF	**−0.557**	**0.079**	**−7.03**	**<0.0001**
TimePoint*SI_1mV_	**0.005**	**0.001**	**5.45**	**<0.0001**
BDNF*SI_1mV_	−0.005	0.019	−0.28	0.7814
TimePoint*BDNF*SI_1mV_	**0.012**	**0.001**	**7.93**	**<0.0001**
Random effects	*s*^2^		
Participants	0.21			

*Note. Significant simple effects and interactions are highlighted in bold. Coef., coefficient; SE, standard error; t, *t*-value; *s*^2^, random effect variance; SI_1mV_, stimulation intensity [defined as percentage of maximum stimulator output (MSO)]. Reference for BDNF is Val66Val*.

*Post hoc* comparisons evaluating the effect of SI within each BDNF genotype group revealed that SI affected the degree to which MEP amplitudes differed from baseline to each post-cTBS timepoint for both Val66Val and Val66Met carriers. Specifically, the extent to which cTBS suppressed MEPs decreased as a function of increasing SI up to 30 min post-cTBS. Moreover, comparisons between BDNF genotype groups at the median of the upper and lower tercile of the range of SI values revealed no difference in the change in MEPs across timepoint for lower SI values (SI_44_; *p* > 0.21), but a significant difference across timepoint for higher SI values (SI_65_; *p* < 0.0001), with Val66Met carriers exhibiting (paradoxical) MEP-facilitation with test pulses delivered at higher SIs. Summary statistics for each *post hoc* comparison are reported in Tables s 4, 5.

**Table 4:** *Post hoc* comparison results assessing the change in MEPs from baseline to post-cTBS time points within each BDNF genotype group as a function of SI.

	*Estimate*	*SE*	*z*	*p*
Val66Val				
**Baseline—Post-cTBS 0 min**	**−0.012**	**0.004**	**−3.27**	**0.0094**
**Baseline—Post-cTBS 10 min**	**−0.019**	**0.004**	**−5.12**	**<0.0001**
**Baseline—Post-cTBS 20 min**	**−0.026**	**0.004**	**−7.11**	**<0.0001**
**Baseline—Post-cTBS 30 min**	**−0.016**	**0.004**	**−4.31**	**0.0002**
Val66Met				
**Baseline—Post-cTBS 0 min**	**−0.041**	**0.005**	**−8.23**	**<0.0001**
**Baseline—Post-cTBS 10 min**	**−0.037**	**0.005**	**−7.44**	**<0.0001**
**Baseline—Post-cTBS 20 min**	**−0.065**	**0.005**	**−13.21**	**<0.0001**
**Baseline—Post-cTBS 30 min**	**−0.068**	**0.006**	**−11.80**	**<0.0001**

*Note. Significant differences in MEP amplitudes for Val66Val and Val66Met carriers are highlighted in bold. Estimate, difference in model-estimated MEPs from Baseline to Post-cTBS time points within each BDNF genotype group (Val66Val and Val66Met); SE, standard error of the estimate; df, degrees of freedom; z, z-value. Statistical test results represent Tukey-adjusted values correcting for multiple comparisons within the family of estimates compared*.

**Table 5:** *Post hoc* comparison results assessing the difference in MEPs across the time points for Val66Val vs. Val66Met BDNF genotypes at the median of the upper (SI_65_) and lower (SI_44_) tercile of the range of SI values.

	*Estimate*	*SE*	*z*	*p*
Contrast				
**Val66Val SI_44_ - Val66Val SI_65_**	**−0.096**	**0.018**	**−5.45**	**<0.0001**
Val66Val SI_44_ - Val66Met SI_44_	0.036	0.019	1.94	0.2113
**Val66Val SI_44_ - Val66Met SI_65_**	**−0.308**	**0.025**	**−12.59**	**<0.0001**
**Val66Val SI_65_ - Val66Met SI_44_**	**0.132**	**0.017**	**7.67**	**<0.0001**
**Val66Val SI_65_ - Val66Met SI_65_**	**−0.213**	**0.024**	**−9.06**	**<0.0001**
**Val66Met SI_44_ - Val66Met SI_65_**	**−0.344**	**0.026**	**−13.26**	**<0.0001**

*Note. Significant differences in MEP amplitudes for Val66Val vs. Val66Met carriers are highlighted in bold. Estimate = difference in model-estimated MEPs for the Upper and Lower SI values (SI_65_ and SI_44_, respectively) across the slope of TimePoint for Val66Val vs. Val66Met BDNF genotypes; SE, standard error of the estimate; df, degrees of freedom; z, z-value. Statistical test results represent Tukey-adjusted values correcting for multiple comparisons within the family of estimates compared*.

**Figure 2:**
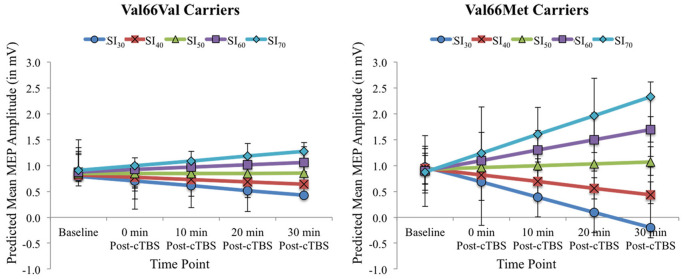
Model-estimated (predicted) MEP amplitudes (in mV) as a function of Time Point (Baseline vs. 0, 10, 20, and 30 post-cTBS) and SI_1mV_ (expressed as the percentage of maximum stimulator output [MSO] separated into 10% increments across the range of SI_1mV_ values as depicted in different colors) for Val66Val (left) and Val66Met carriers (right). Error bars represent 95% confidence intervals.

Figure 2 displays the full model predicted estimates of MEPs for each timepoint as a function of SI for each BDNF genotype group. Estimates were extracted from the model using the effects package in R (Fox and Weisberg, 2018), which yielded separate estimates per 10% increments of total percentage MSO across the range SI_1mV_ values for the two BDNF genotype groups and each timepoint. The model-estimated MEPs pre- and post-cTBS reflect predicted values after accounting for the covariate age and inter-individual variability in MEPs prior to cTBS (represented in the by-participant random intercept). As shown in Figure 2, the present findings demonstrate that test pulse SI has a differential impact on cTBS aftereffects among BDNF Val66Val carriers when compared to Val66Met carriers. While increasing SI attenuated cTBS-induced MEP suppression for both BDNF genotype groups, this effect was more pronounced for Val66Met carriers, as higher test pulse SIs yielded paradoxical (excitatory) cTBS responses from baseline to post-cTBS timepoints raw mean MEPs per SI interval for Val66Val vs. Val66Met carriers (corresponding to the predicted values depicted in Figure 2) can be found in Supplementary Table 2.

## Discussion

This study reports two key findings important for understanding potential sources of variability in cTBS response. First, SI impacted cTBS aftereffects, with both BDNF genotype groups exhibiting less MEP suppression from baseline to post-cTBS timepoints with higher SIs. Second, SI used to probe cortical excitability before and after cTBS accounted for variable cTBS aftereffects among Met allele carriers to a greater extent than their homozygous (Val66Val) counterparts. Thus, our data confirm and extend existing results demonstrating that inter-subject variability in response to cTBS arises due to intrinsic (genetic) factors (Cheeran et al., 2008; Mastroeni et al., 2013; Jannati et al., 2017) and extrinsic (methodologic) factors (Vallence et al., 2015; Goldsworthy et al., 2016b), and indicates that these two sources of variability interact in meaningful ways.

To date, only a few studies have systematically investigated whether BDNF Val66Met genotype status affects the response to cTBS (Cheeran et al., 2008; Mastroeni et al., 2013; Jannati et al., 2017). However, the findings from these studies have been mixed, precluding any definitive conclusions regarding whether BDNF genotype status contributes to inter-individual differences in cTBS response more generally. This suggests that cTBS response is more variable among Met allele carriers when compared to their homozygous (Val66Val) counterparts (Chung et al., 2016). Here, we demonstrated that the SI used to probe cortical excitability predicted both the magnitude and direction of cTBS-induced changes in plasticity for Met allele carriers to a greater extent than Val allele carriers, which provides a framework for understanding prior equivocal results *and* variability in cTBS response. Across the studies using different methods for determining SI to probe cTBS-induced plasticity, SIs evoking 0.5 mV yielded null effects of BDNF genotype status (Mastroeni et al., 2013), SI_1mV_ yielded little-to-no changes in cortical excitability for Met allele carriers (Cheeran et al., 2008), and SI at 120% rMT (evoking on average MEPs of 1.4 mV) yielded paradoxical effects of cTBS (MEP facilitation) for Met allele carriers (Jannati et al., 2017). When considered alongside the findings from the current study, this suggests that for Met allele carriers, cTBS becomes increasingly facilitatory with increasing test pulse intensity, especially when compared to homozygous Val allele carriers, which is consistent with the hypothesis that polymorphisms in the BDNF genotype constitute an important source of inter-individual variability in cTBS responsiveness.

Inconsistencies regarding the impact of BDNF polymorphisms on stimulation-induced neuroplasticity are not unique to cTBS protocols, as both significant and null effects of BDNF genotype status have been obtained in studies using several different noninvasive brain stimulation techniques, such as iTBS (Antal et al., 2010; Lee et al., 2013), paired associative stimulation (PAS; Cheeran et al., 2008; Witte et al., 2012), and cathodal tDCS (Cheeran et al., 2008; Antal et al., 2010). However, whether these discrepant findings also reflect methodological differences in the SI used to probe plasticity remains unclear. As in the current research, these studies assessed changes in cortical excitability using SI_1mV_—a method that necessarily introduces variability as it pertains to the degree to which SI exceeds a given individual’s resting motor threshold in order to attain MEPs of ~1 mV. However, to our knowledge, no prior study has explored whether variability in the intensity required to achieve MEPs of 1 mV at baseline, i.e., prior to repetitive stimulation, interacts with BDNF genotype status. If true, our results predict that variability in SI among each study’s sample would explain why Met allele carriers as a group either exhibit the expected pattern of response or reduced and/or paradoxical responses to plasticity-inducing noninvasive brain stimulation techniques. Future research is needed to fully explore this hypothesis.

It is important to note, however, that our finding of decreased MEP-suppression (and increasing MEP-facilitation among Met allele carriers) with increasing SI is not consistent with prior research exploring cTBS aftereffects across individuals’ input-output (IO) curves. That is, evidence elsewhere demonstrates that cTBS-induced MEP-suppression is greatest with SIs ~150% of rMT (Vallence et al., 2015; Goldsworthy et al., 2016b). However, these studies differ from the current research in two potentially important ways. First, cTBS intensity was determined using rMT, whereas the present research used aMT to determine cTBS intensity. Given that prior activation of the targeted muscle has been shown to affect both the magnitude and direction of cTBS aftereffects (Gentner et al., 2007; Iezzi et al., 2008), it is possible that this methodological difference also contributes to variability in cTBS response. Second, these studies did not investigate potential differences in SI as a function of BDNF genotype status, leaving it an open question as to whether or not pre-active vs. pre-relax muscle contraction would represent an additional source of variability depending on this genetic factor. Thus, it will be important to explore other potential interactions between genetic and experimentally-imposed sources of cTBS response variability in future work.

Why is there facilitation of MEPs, rather than the expected MEP-suppression, following cTBS among Val66Met carriers at higher SIs? While the precise mechanisms underlying this effect are beyond the scope of the current study, findings from prior work provide future avenues of study. It has been shown that the increase in the percentage of MSO required to produce the full range of possible MEP amplitudes can vary considerably across individuals and that both intrinsic and external factors contribute to this variability (Goetz et al., 2014). Evidence elsewhere suggests that test pulse SI along the IO curve impacts response to plasticity-inducing protocols (Vallence et al., 2015; Goldsworthy et al., 2016b). Moreover, some evidence suggests that, depending on whether the initial response to cTBS is in the predicted direction, increasing or decreasing cTBS intensity can reverse the direction of the initial response (Sasaki et al., 2018). In other words, the aftereffects of plasticity-inducing protocols like cTBS may vary as a function of both the intensity at which stimulation is administered and the intensity at which the cortex is probed before and following modulation. Our findings add further to this complexity by suggesting that the IO curve of Met allele carriers may inherently differ from that of homozygous Val allele carriers, which in turn affects the direction of their response to plasticity-inducing protocols like cTBS. Partial support for this hypothesis comes from studies that have explored whether rTMS intensity interacts with BDNF genotype status (Hwang et al., 2015; also see Jannati et al., 2017 for evidence indicative of a potential interaction between cTBS intensity and BDNF genotype status). We suggest that future studies investigate the ways in which stimulation protocols may influence response to plasticity-inducing protocols, and more importantly the potential interaction between methodological choices and BDNF genotype status. This may yield additional promising insights into the sources of variable rTMS response and in turn, the neurophysiology that dictates said variability.

There are a few limitations to our study. First, the sample size is relatively small, which may affect the generalizability of our results. However, several factors may militate against this concern. Our sample size is: (1) comparable to prior studies investigating the effects of BDNF polymorphisms on stimulation-induced changes in neuroplasticity—whether reporting positive (Cheeran et al., 2008) or negative results (Mastroeni et al., 2013); and (2) representative of the occurrence of BDNF polymorphisms in the population (Shimizu et al., 2004). Critically, the analysis approach adopted here is relatively robust to biases in estimated effects for between-subject factors with unequal sample sizes (Maas and Hox, 2005; Bell et al., 2014). This is because linear mixed effects modeling can use trial-level data, which captures variability between subjects prior to the intervention. We argue that this represents a major advantage over prior statistical methods that have obscured inter- and intra-individual differences by analyzing changes in an individual’s mean MEPs across a block of trials, which may be contaminated by cumulative effects (Pellicciari et al., 2016) and/or transforming MEP values to reflect a percentage change in mean/maximum MEP values obtained at baseline (e.g., Sasaki et al., 2018). Second, SI used to probe plasticity varied across individuals, rather than within individuals. Thus, it will be important for future work to confirm and extend these findings by comparing the IO curves of individuals with and without the BDNF polymorphism as it relates to neuroplastic responses to rTMS protocols. Finally, it could be argued that the findings reported here were not specific to cTBS, as the current study did not include a sham-control arm—as is the case in the majority of studies investigating stimulation-induced motor excitability (e.g., Cheeran et al., 2008; Vallence et al., 2015; Goldsworthy et al., 2016b; Jannati et al., 2017, 2019). However, we believe the lack of a sham-control condition does not undermine the main findings. Prior work that has included a sham-control arm, but did not investigate genetic moderators of rTMS response, demonstrates that cortical excitability changes in the expected direction following real, but not sham, stimulation (e.g., Todd et al., 2009; Albuquerque et al., 2018), suggesting that the effects of rTMS on MEP amplitudes are not due to order effects (i.e., prior elicitation of MEPs *via* single-pulse TMS) and/or placebo effects. Regarding the BDNF polymorphism, studies have shown that MEPs decrease to a greater extent for homozygous Val carriers when compared to Val66Met carriers in the absence of repetitive NIBS protocols (e.g., motor learning tasks; e.g., Kleim et al., 2006); however, the current study did not include a voluntary motor (learning) component. Thus, we would expect similar changes in MEPs from pre- to post-cTBS for both BDNF genotype groups if the findings were not specific to the stimulation itself. Nonetheless, it is still important to replicate and extend these findings in a sham-controlled study with a larger sample size sampled across the IO curve.

### Conclusions

Overall, our data provide novel insight into the sources of variability in response to rTMS protocols, which has important implications for optimizing the clinical utility of this neurorehabilitative tool. The finding that SI used to probe plasticity modulated cTBS response for Met allele carriers to a greater extent than their homozygous (Val66Val) counterparts indicates that genetic factors interact with methodological sources of variability. Given that these two sources of variability interact in a meaningful way, it is important that future work take this into consideration. Further refinement of our understanding of the complex interplay between stimulation parameters that determine the effects of TMS and biologically-based individual factors that influence neuroplasticity may allow for further optimization and personalization of both experimental and therapeutic brain stimulation protocols.

## Data Availability Statement

The raw data supporting the conclusions of this article will be made available by the authors, without undue reservation.

## Ethics Statement

The studies involving human participants were reviewed and approved by Institutional Review Board of the University of Pennsylvania. The patients/participants provided their written informed consent to participate in this study.

## Author Contributions

RH, PS-B, and OF contributed to the design of the study. DH, LD, PS-B, RW, DS, and OF collected the data. DH, LD, PS-B, RW, DS, AA, AT, and OF assisted with pre-processing and analysis of neurophysiological data. FL performed genotyping of saliva samples. DH and LD analyzed and interpreted the full dataset and drafted the manuscript. All authors revised the manuscript, approved the final version, and agreed to be accountable for the content of the work. All authors contributed to the article and approved the submitted version.

## Conflict of Interest

The authors declare that the research was conducted in the absence of any commercial or financial relationships that could be construed as a potential conflict of interest.

## Footnotes

^1^Note that the results did not change when analyses were conducted on untrimmed means.

## References

[B1] Albuquerque P. L. Campêlo M. Mendonça T. Fontes L. A. M. Brito R. D. M. Monte-Silva K. (2018). Effects of repetitive transcranial magnetic stimulation and trans-spinal direct current stimulation associated with treadmill exercise in spinal cord and cortical excitability of healthy subjects: a triple-blind, randomized and sham-controlled study. PLoS One 13:e0195276. 10.1371/journal.pone.0195276 29596524 PMC5875883

[B2] Antal A. Chaieb L. Moliadze V. Monte-Silva K. Poreisz C. Thirugnanasambandam N.. (2010). Brain-derived neurotrophic factor (BDNF) gene polymorphisms shape cortical plasticity in humans. Brain Stimul. 3, 230–237. 10.1016/j.brs.2009.12.003 20965453

[B3] Baayen R. H. Davidson D. J. Bates D. M. (2008). Mixed-effects modeling with crossed random effects for subjects and items. J. Mem. Lang. 59, 390–412. 10.1080/00273171.2021.1889946

[B4] Barr D. J. Levy R. Scheepers C. Tily H. J. (2013). Random effects structure for confirmatory hypothesis testing: keep it maximal. J. Mem. Lang. 68, 255–278. 10.1016/j.jml.2012.11.001 24403724 PMC3881361

[B5] Bates D. Maechler M. Bolker B. Walker S. (2015). Fitting linear mixed-effects models using lme4. J. Stat. Softw. 67, 1–48. 10.18637/jss.v067.i01

[B6] Bath K. G. Lee F. S. (2006). Variant BDNF (Val66Met) impact on brain structure and function. Cogn. Affect. Behav. Neurosci. 6, 79–85. 10.3758/cabn.6.1.79 16869232

[B7] Bell B. A. Morgan G. B. Schoeneberger J. A. Kromrey J. D. Ferron J. M. (2014). How low can you go? An investigation of the influence of sample size and model complexity on point and interval estimates in two-level linear models. Methodology 10:1. 10.1027/1614-2241/a000062

[B8] Bolognini N. Pascual-Leone A. Fregni F. (2009). Using non-invasive brain stimulation to augment motor training-induced plasticity. J. Neuroeng. Rehabil. 6:8. 10.1186/1743-0003-6-8 19292910 PMC2667408

[B9] Cheeran B. Talelli P. Mori F. Koch G. Suppa A. Edwards M.. (2008). A common polymorphism in the brain-derived neurotrophic factor gene (BDNF) modulates human cortical plasticity and the response to rTMS. J. Physiol. 586, 5717–5725. 10.1113/jphysiol.2008.159905 18845611 PMC2655403

[B10] Chung S. W. Hill A. T. Rogasch N. C. Hoy K. E. Fitzgerald P. B. (2016). Use of theta-burst stimulation in changing excitability of motor cortex: a systematic review and meta-analysis. Neurosci. Biobehav. Rev. 63, 43–64. 10.1016/j.neubiorev.2016.01.008 26850210

[B11] Connolly K. R. Helmer A. Cristancho M. A. Cristancho P. O’Reardon J. P. (2012). Effectiveness of transcranial magnetic stimulation in clinical practice post-FDA approval in the United States: results observed with the first 100 consecutive cases of depression at an academic medical center. J. Clin. Psychiatry 73, e567–e573. 10.4088/JCP.11m07413 22579164

[B12] Corp D. T. Bereznicki H. G. Clark G. M. Youssef G. J. Fried P. J. Jannati A.. (2020). Large-scale analysis of interindividual variability in theta-burst stimulation data: results from the ‘Big TMS data collaboration’. Brain Stimul. 13, 1476–1488. 10.1016/j.brs.2020.07.018 32758665 PMC7494610

[B13] Devlin J. T. Watkins K. E. (2006). Stimulating language: insights from TMS. Brain 130, 610–622. 10.1093/brain/awl331 17138570 PMC1820607

[B14] Egan M. F. Kojima M. Callicott J. H. Goldberg T. E. Kolachana B. S. Bertolino A.. (2003). The BDNF val66met polymorphism affects activity-dependent secretion of BDNF and human memory and hippocampal function. Cell 112, 257–269. 10.1016/s0092-8674(03)00035-7 12553913

[B15] Era V. Aglioti S. M. Candidi M. (2020). Inhibitory theta burst stimulation highlights the role of left aIPS and right TPJ during complementary and imitative human-avatar interactions in cooperative and competitive scenarios. Cereb. Cortex 30, 1677–1687. 10.1093/cercor/bhz195 31667496

[B16] Era V. Candidi M. Gandolfo M. Sacheli L. M. Aglioti S. M. (2018). Inhibition of left anterior intraparietal sulcus shows that mutual adjustment marks dyadic joint-actions in humans. Soc. Cogn. Affect. Neurosci. 13, 492–500. 10.1093/scan/nsy022 29660090 PMC6007351

[B17] Ferrari C. Vecchi T. Todorov A. Cattaneo Z. (2016). Interfering with activity in the dorsomedial prefrontal cortex *via* TMS affects social impressions updating. Cogn. Affect. Behav. Neurosci. 16, 626–634. 10.3758/s13415-016-0419-2 27012713

[B18] Field A. P. Wright D. B. (2011). A primer on using multilevel models in clinical and experimental psychopathology research. J. Exp. Psychopathol. 2, 271–293. 10.1016/j.brat.2017.05.013 28577757

[B19] Fini C. Fischer M. Bardi L. Brass M. Moors A. (2020). Support from a TMS/MEP study for a direct link between positive/negative stimuli and approach/avoidance tendencies. Neuropsychologia 143:107496. 10.1016/j.neuropsychologia.2020.107496 32407905

[B20] Fox J. Weisberg S. (2018). An R Companion to Applied Regression. 3rd Edn. Thousand Oaks, CA: Sage Publications.

[B21] Freitas C. Perez J. Knobel M. Tormos J. M. Oberman L. M. Eldaief M.. (2011). Changes in cortical plasticity across the lifespan. Front. Aging Neurosci. 3:5. 10.3389/fnagi.2011.00005 21519394 PMC3079175

[B22] Gentner R. Wankerl K. Reinsberger C. Zeller D. Classen J. (2007). Depression of human corticospinal excitability induced by magnetic theta-burst stimulation: evidence of rapid polarity-reversing metaplasticity. Cereb. Cortex 18, 2046–2053. 10.1093/cercor/bhm239 18165282

[B23] Goetz S. M. Luber B. Lisanby S. H. Murphy D. L. Kozyrkov I. C. Grill W. M.. (2016). Enhancement of neuromodulation with novel pulse shapes generated by controllable pulse parameter transcranial magnetic stimulation. Brain Stimul. 9, 39–47. 10.1016/j.brs.2015.08.013 26460199 PMC5517314

[B24] Goetz S. M. Luber B. Lisanby S. H. Peterchev A. V. (2014). A novel model incorporating two variability sources for describing motor evoked potentials. Brain Stimul. 7, 541–552. 10.1016/j.brs.2014.03.002 24794287 PMC4108579

[B25] Goldsworthy M. R. Hordacre B. Ridding M. C. (2016a). Minimum number of trials required for within-and between-session reliability of TMS measures of corticospinal excitability. Neuroscience 320, 205–209. 10.1016/j.neuroscience.2016.02.012 26872998

[B27] Goldsworthy M. R. Vallence A. M. Hodyl N. A. Semmler J. G. Pitcher J. B. Ridding M. C. (2016b). Probing changes in corticospinal excitability following theta burst stimulation of the human primary motor cortex. Clin. Neurophysiol. 127, 740–747. 10.1016/j.clinph.2015.06.014 26122069

[B26] Goldsworthy M. R. Müller-Dahlhaus F. Ridding M. C. Ziemann U. (2014). Inter-subject variability of LTD-like plasticity in human motor cortex: a matter of preceding motor activation. Brain Stimul. 7, 864–870. 10.1016/j.brs.2014.08.004 25216649

[B28] Harrison X. A. Donaldson L. Correa-Cano M. E. Evans J. Fisher D. N. Goodwin C. E.. (2018). A brief introduction to mixed effects modelling and multi-model inference in ecology. PeerJ 6:e4794. 10.7717/peerj.4794 29844961 PMC5970551

[B29] Hordacre B. Goldsworthy M. R. Vallence A. M. Darvishi S. Moezzi B. Hamada M.. (2017). Variability in neural excitability and plasticity induction in the human cortex: a brain stimulation study. Brain Stimul. 10, 588–595. 10.1016/j.brs.2016.12.001 28024963

[B30] Huang E. J. Reichardt L. F. (2001). Neurotrophins: roles in neuronal development and function. Annu. Rev. Neurosci. 24, 677–736. 10.1146/annurev.neuro.24.1.677 11520916 PMC2758233

[B31] Huang Y. Z. Edwards M. J. Rounis E. Bhatia K. P. Rothwell J. C. (2005). Theta burst stimulation of the human motor cortex. Neuron 45, 201–206. 10.1016/j.neuron.2004.12.033 15664172

[B32] Hwang J. M. Kim Y. H. Yoon K. J. Uhm K. E. Chang W. H. (2015). Different responses to facilitatory rTMS according to BDNF genotype. Clin. Neurophysiol. 126, 1348–1353. 10.1016/j.clinph.2014.09.028 25454277

[B33] Iezzi E. Conte A. Suppa A. Agostino R. Dinapoli L. Scontrini A.. (2008). Phasic voluntary movements reverse the aftereffects of subsequent theta-burst stimulation in humans. J. Neurophysiol. 100, 2070–2076. 10.1152/jn.90521.2008 18753328

[B34] Jannati A. Block G. Oberman L. M. Rotenberg A. Pascual-Leone A. (2017). Interindividual variability in response to continuous theta-burst stimulation in healthy adults. Clin. Neurophysiol. 128, 2268–2278. 10.1016/j.clinph.2017.08.023 29028501 PMC5675807

[B35] Jannati A. Fried P. J. Block G. Oberman L. M. Rotenberg A. Pascual-Leone A. (2019). Test-retest reliability of the effects of continuous theta-burst stimulation. Front. Neurosci. 13:447. 10.3389/fnins.2019.00447 31156361 PMC6533847

[B36] Kleim J. A. Chan S. Pringle E. Schallert K. Procaccio V. Jimenez R.. (2006). BDNF val66met polymorphism is associated with modified experience-dependent plasticity in human motor cortex. Nat. Neurosci. 9, 735–737. 10.1038/nn1699 16680163

[B37] Lee M. Kim S. E. Kim W. S. Lee J. Yoo H. K. Park K. D.. (2013). Interaction of motor training and intermittent theta burst stimulation in modulating motor cortical plasticity: influence of BDNF Val66Met polymorphism. PLoS One 8:e57690. 10.1371/journal.pone.0057690 23451258 PMC3581515

[B38] Lenth R. (2020). emmeans: Estimated Marginal Means, aka Least-Squares Means. R package version 1.5.2–1. Available online at: https://CRAN.R-project.org/package=emmeans.

[B39] Li Voti P. Conte A. Suppa A. Iezzi E. Bologna M. Aniello M. S.. (2011). Correlation between cortical plasticity, motor learning and BDNF genotype in healthy subjects. Exp. Brain Res. 212, 91–99. 10.1007/s00221-011-2700-5 21537966

[B40] Lowe C. J. Manocchio F. Safati A. B. Hall P. A. (2018). The effects of theta burst stimulation (TBS) targeting the prefrontal cortex on executive functioning: a systematic review and meta-analysi. Neuropsychologia 111, 344–359. 10.1016/j.neuropsychologia.2018.02.004 29438672

[B41] Lu B. (2003). BDNF and activity-dependent synaptic modulation. Learn. Mem. 10, 86–98. 10.1101/lm.54603 12663747 PMC5479144

[B42] Maas C. J. Hox J. J. (2005). Sufficient sample sizes for multilevel modeling. Methodology 1:86. 10.1027/1614-2241.1.3.86

[B43] Mastroeni C. Bergmann T. O. Rizzo V. Ritter C. Klein C. Pohlmann I.. (2013). Brain-derived neurotrophic factor-a major player in stimulation-induced homeostatic metaplasticity of human motor cortex? PloS One 8:e57957. 10.1371/journal.pone.0057957 23469118 PMC3585283

[B44] McHughen S. A. Rodriguez P. F. Kleim J. A. Kleim E. D. Crespo L. M. Procaccio V.. (2009). BDNF val66met polymorphism influences motor system function in the human brain. Cereb. Cortex 20, 1254–1262. 10.1093/cercor/bhp189 19745020 PMC2852510

[B45] Medaglia J. D. Harvey D. Y. White N. Kelkar A. Zimmerman J. Bassett D. S.. (2018). Network controllability in the inferior frontal gyrus relates to controlled language variability and susceptibility to TMS. J. Neurosci. 38, 6399–6410. 10.1523/JNEUROSCI.0092-17.2018 29884739 PMC6041793

[B46] Mochizuki H. Huang Y. Z. Rothwell J. C. (2004). Interhemispheric interaction between human dorsal premotor and contralateral primary motor cortex. J. Physiol. 561, 331–338. 10.1113/jphysiol.2004.072843 15459244 PMC1665328

[B47] Moors A. Fini C. Everaert T. Bardi L. Bossuyt E. Kuppens P.. (2019). The role of stimulus-driven versus goal-directed processes in fight and flight tendencies measured with motor evoked potentials induced by transcranial magnetic stimulation. PloS One 14:e0217266. 10.1371/journal.pone.0217266 31107906 PMC6527228

[B48] Moret B. Donato R. Nucci M. Cona G. Campana G. (2019). Transcranial random noise stimulation (tRNS): a wide range of frequencies is needed for increasing cortical excitability. Sci. Rep. 9, 1–8. 10.1038/s41598-019-51553-7 31641235 PMC6806007

[B49] Ninan I. Bath K. G. Dagar K. Perez-Castro R. Plummer M. R. Lee F. S.. (2010). The BDNF Val66Met polymorphism impairs NMDA receptor-dependent synaptic plasticity in the hippocampus. J. Neurosci. 30, 8866–8870. 10.1523/JNEUROSCI.1405-10.2010 20592208 PMC2911131

[B50] Pascual-Leone A. Tormos J. M. Keenan J. Tarazona F. Cañete C. Catalá M. D. (1998). Study and modulation of human cortical excitability with transcranial magnetic stimulation. J. Clin. Neurophysiol. 15, 333–343. 10.1097/00004691-199807000-00005 9736467

[B51] Pellicciari M. C. Miniussi C. Ferrari C. Koch G. Bortoletto M. (2016). Ongoing cumulative effects of single TMS pulses on corticospinal excitability: An intra-and inter-block investigation. Clin. Neurophysiol. 127, 621–628. 10.1016/j.clinph.2015.03.002 25823698

[B500] R Core Team. (2020). The R Project for Statistical Computing. Vienna, Austria: The R Foundation. Available online at: https://cran.r-project.org/bin/windows/base/old/3.6.3/.

[B52] Ridding M. C. Ziemann U. (2010). Determinants of the induction of cortical plasticity by non-invasive brain stimulation in healthy subjects. J. Physiol. 588, 2291–2304. 10.1113/jphysiol.2010.190314 20478978 PMC2915507

[B53] Rossini P. M. Berardelli A. Deuschl G. Hallett M. Maertens de Noordhout A. M. Paulus W.. (1999). Applications of magnetic cortical stimulation. The international federation of clinical neurophysiology. Electroencephalogr. Clin. Neurophysiol. 52, 171–185. 10590986

[B54] Rothwell J. C. Hallett M. Berardelli A. Eisen A. Rossini P. Paulus W. (1999). Magnetic stimulation: Motor evoked potentials. The international federation of clinical neurophysiology. Electroencephalogr. Clin. Neurophysiol. 52, 97–103. 10590980

[B55] Sasaki T. Kodama S. Togashi N. Shirota Y. Sugiyama Y. Tokushige S. I.. (2018). The intensity of continuous theta burst stimulation, but not the waveform used to elicit motor evoked potentials, influences its outcome in the human motor cortex. Brain Stimul. 11, 400–410. 10.1016/j.brs.2017.12.003 29258807

[B56] Schlaepfer T. E. George M. S. Mayberg H. (2010). WFSBP task force on brain stimulation. WFSBP guidelines on brain stimulation treatments in psychiatry. World J. Biol. Psychiatry 11, 2–18. 10.3109/15622970903170835 20146648

[B57] Schwedt T. J. Vargas B. (2015). Neurostimulation for treatment of migraine and cluster headache. Pain Med. 16, 1827–1834. 10.1111/pme.12792 26177612 PMC4572909

[B58] Shimizu E. Hashimoto K. Iyo M. (2004). Ethnic difference of the BDNF 196G/A (val66met) polymorphism frequencies: the possibility to explain ethnic mental traits. Am. J. Med. Genet. B Neuropsychiatr. Genet. 126, 122–123. 10.1002/ajmg.b.20118 15048661

[B59] Soliman F. Glatt C. E. Bath K. G. Levita L. Jones R. M. Pattwell S. S.. (2010). A genetic variant BDNF polymorphism alters extinction learning in both mouse and human. Science 327, 863–866. 10.1126/science.1181886 20075215 PMC2829261

[B60] Todd G. Rogasch N. C. Flavel S. C. Ridding M. C. (2009). Voluntary movement and repetitive transcranial magnetic stimulation over human motor cortex. J. Appl. Physiol. 106, 1593–1603. 10.1152/japplphysiol.91364.2008 19246656

[B61] Vallence A. M. Goldsworthy M. R. Hodyl N. A. Semmler J. G. Pitcher J. B. Ridding M. C. (2015). Inter-and intra-subject variability of motor cortex plasticity following continuous theta-burst stimulation. Neuroscience 304, 266–278. 10.1016/j.neuroscience.2015.07.043 26208843

[B62] Voelker R. (2018). Brain stimulation approved for obsessive-compulsive disorder. JAMA 320:1098. 10.1001/jama.2018.13301 30422286

[B63] Warrington N. M. Tilling K. Howe L. D. Paternoster L. Pennell C. E. Wu Y. Y.. (2014). Robustness of the linear mixed effects model to error distribution assumptions and the consequences for genome-wide association studies. Stat. Appl. Genet. Mol. Biol. 13, 567–587. 10.1515/sagmb-2013-0066 25153607

[B64] Witte A. V. Kürten J. Jansen S. Schirmacher A. Brand E. Sommer J.. (2012). Interaction of BDNF and COMT polymorphisms on paired-associative stimulation-induced cortical plasticity. J. Neurosci. 32, 4553–4561. 10.1523/JNEUROSCI.6010-11.2012 22457502 PMC6622078

[B65] Zuur A. F. Ieno E. N. Elphick C. S. (2010). A protocol for data exploration to avoid common statistical problems. Methods Ecol. Evol. 1, 3–14. 10.1111/j.2041-210X.2009.00001.x

