# Supplementary material for: Variability in cTBS Aftereffects Attributed to the Interaction of Stimulus Intensity With BDNF Val66Met Polymorphism
Source: Front Hum Neurosci. 2021 Jun 18;15:585533. doi: 10.3389/fnhum.2021.585533 (PMC8249815; doi:10.3389/fnhum.2021.585533)
Supplement: Supplementary file 1 [file Table_1.DOCX]

# Supplementary Table 1.

*Participant Demographic Information and Stimulation Parameters.*

| **Subj ID** | **BDNF Genotype** | **Race** | **Age** | **Sex** | **rMT** | **SI_1mV_** | **% rMT** | **Mean MEP: Baseline** | **Mean MEP: 0 Min Post-cTBS** | **Mean MEP: 10 Min Post-cTBS** | **Mean MEP: 20 Min Post-cTBS** | **Mean MEP: 30 Min Post-cTBS** |
| --- | --- | --- | --- | --- | --- | --- | --- | --- | --- | --- | --- | --- |
| S03 | Val66Met | Asian | 26 | M | 45 | 52 | 115.6 | 0.574 | 0.219 | 0.59 | 0.66 | 0.48 |
| S06 | Val66Met | Caucasian | 39 | F | 31 | 47 | 151.6 | 0.507 | 0.407 | 0.45 | 0.61 | NA |
| S13 | Val66Met | Caucasian | 35 | M | 30 | 32 | 106.7 | 0.484 | 0.958 | 1.12 | 0.66 | NA |
| S14 | Val66Met | Asian | 19 | M | 49 | 53 | 108.2 | 0.691 | 0.967 | 1.12 | 1.14 | 1.25 |
| S15 | Val66Met | African American | 21 | F | 42 | 56 | 133.3 | 0.997 | 1.289 | 1.79 | 0.98 | 1.83 |
| S17 | Val66Met | Caucasian/Hispanic | 33 | M | 42 | 54 | 128.6 | 0.463 | 0.726 | 0.51 | 0.51 | 0.66 |
| S23 | Val66Met | Caucasian/Hispanic | 19 | M | 49 | 64 | 130.6 | 1.403 | 3.261 | 2.71 | 4.50 | 3.67 |
| S27 | Val66Met | Asian | 22 | F | 41 | 46 | 112.2 | 0.862 | 0.253 | 0.84 | 0.43 | 0.20 |
| S28 | Val66Met | Caucasian | 21 | M | 53 | 56 | 105.7 | 0.637 | 0.470 | 0.42 | 0.57 | 0.45 |
| S29 | Val66Met | Caucasian | 22 | M | 46 | 52 | 113.0 | 1.069 | 0.293 | 0.45 | 0.53 | 0.57 |
| S30 | Val66Met | Caucasian | 23 | F | 30 | 35 | 116.7 | 1.806 | 0.864 | 0.48 | 0.88 | 0.80 |
| S01 | Val66Val | African American | 23 | F | 43 | 57 | 132.6 | 1.284 | 1.107 | 0.93 | 0.95 | 0.51 |
| S02 | Val66Val | African American | 19 | F | 55 | 66 | 120.0 | 0.979 | 1.304 | 0.88 | 1.51 | 1.35 |
| S04 | Val66Val | NA | 21 | F | 53 | 56 | 105.7 | 0.984 | 0.314 | 0.62 | 0.79 | 0.62 |
| S05 | Val66Val | Caucasian/Asian | 22 | M | 35 | 44 | 125.7 | 0.864 | 0.708 | 0.84 | 0.78 | 1.09 |
| S07 | Val66Val | African American | 19 | F | 37 | 43 | 116.2 | 1.669 | 0.856 | 1.06 | 1.00 | 0.98 |
| S08 | Val66Val | African American | 21 | M | 56 | 66 | 117.9 | 1.515 | 0.708 | 0.36 | 0.37 | 0.34 |
| S09 | Val66Val | African American | 22 | M | 42 | 49 | 116.7 | 1.099 | 0.763 | 0.53 | 1.07 | 0.74 |
| S10 | Val66Val | African American | 18 | F | 61 | 71 | 116.4 | 1.306 | 1.364 | 2.36 | 2.88 | 3.09 |
| S11 | Val66Val | Caucasian | 23 | M | 42 | 44 | 104.8 | 0.895 | 0.606 | 0.63 | 0.25 | 1.10 |
| S12 | Val66Val | NA | 24 | F | 39 | 46 | 117.9 | 0.913 | 0.311 | 0.37 | 0.18 | 1.36 |
| S16 | Val66Val | Caucasian | 25 | F | 44 | 49 | 111.4 | 0.963 | 1.085 | 0.92 | 1.22 | 1.04 |
| S18 | Val66Val | Asian | 22 | M | 56 | 71 | 126.8 | 0.939 | 1.195 | 1.42 | 1.46 | 1.00 |
| S19 | Val66Val | Caucasian | 28 | M | 48 | 61 | 127.1 | 0.898 | 1.009 | 1.12 | 1.15 | 1.58 |
| S20 | Val66Val | Caucasian | 24 | F | 56 | 66 | 117.9 | 0.699 | 0.925 | 0.93 | 0.99 | 0.92 |
| S21 | Val66Val | African American | 22 | F | 64 | 70 | 109.4 | 0.895 | 0.349 | 0.23 | 0.17 | NA |
| S22 | Val66Val | African American | 22 | F | 49 | 57 | 116.3 | 0.724 | 0.447 | 1.02 | 0.52 | 1.40 |
| S24 | Val66Val | African American | 45 | M | 51 | 56 | 109.8 | 0.992 | 0.408 | 0.34 | 0.25 | 0.33 |
| S25 | Val66Val | Caucasian | 26 | M | 38 | 42 | 110.5 | 0.831 | 0.607 | 0.57 | 0.62 | 0.69 |
| S26 | Val66Val | African American | 25 | F | 52 | 57 | 109.6 | 1.174 | 0.846 | 0.75 | 1.24 | 0.71 |
| S31 | Val66Val | Caucasian | 19 | F | 52 | 59 | 113.5 | 0.975 | 0.826 | 1.41 | 1.37 | 1.80 |
| S32* | Met66Met | Asian | 35 | M | 54 | 66 | 122.2 | 0.94 | 0.78 | 0.53 | 0.50 | 0.56 |
| S33* | Met66Met | Caucasian | 28 | M | 33 | 37 | 112.1 | 1.72 | 1.48 | 2.40 | 2.07 | 2.16 |

*Note.* Subjects included in analyses were 31 neurologically healthy individuals (16 females) aged 18-45 (mean (M) ± standard deviation (SD) = 24.2 ± 6.0 years).

*Abbreviations.* Subj ID = subject identification; BDNF = brain-derived neurotropic factor (Val66Val, Val66Met, Met66Met); rMT = resting motor threshold (defined as percentage of maximum stimulator output [MSO]); SI_1mV_ = stimulation intensity (defined as percentage of MSO); mV = millivolt; MEP = motor evoked potential; min = minutes; NA = not available; * = participants not included in analyses due to too few Met66Met allele carriers in the study sample.

# Supplementary Table 2.

*Mean of Raw Motor Evoked Potentials Per SI_1mV_ Interval Separated by BDNF Genotype Status and Time Point.*

|  | Baseline | 0 min Post-cTBS | 10 min Post-cTBS | 20 min Post-cTBS | 30 min Post-cTBS |
| --- | --- | --- | --- | --- | --- |
| Val66Val per SI_1mV_ |  |  |  |  |  |
| SI_30_ | 1.22 | 0.72 | 0.81 | 0.80 | 0.82 |
| SI_40_ | 0.89 | 0.54 | 0.62 | 0.41 | 1.18 |
| SI_50_ | 1.03 | 0.93 | 0.73 | 1.15 | 0.87 |
| SI_60_ | 1.00 | 0.70 | 0.89 | 0.90 | 1.03 |
| SI_70_ | 1.04 | 0.96 | 1.06 | 1.22 | 1.42 |
| Val66Met per SI_1mV_ | |  |  |  |  |
| SI_30_ | 1.22 | 0.90 | 0.79 | 0.78 | 0.80 |
| SI_40_ | 0.72 | 0.33 | 0.66 | 0.51 | 0.20 |
| SI_50_ | 0.73 | 0.53 | 0.68 | 0.73 | 0.74 |
| SI_60_ | 0.80 | 0.83 | 1.05 | 0.74 | 1.07 |
| SI_70_ | 1.40 | 3.26 | 2.71 | 4.50 | 3.67 |

*Note.* Min = minutes; cTBS = continuous theta burst stimulation; SI_30_ = SI_1mV_ ~30% of MSO; SI_40_ = SI_1mV_ ~40% of MSO; SI_50_ = SI_1mV_ ~50% of MSO; SI_60_ = SI_1mV_ ~60% of MSO; SI_70_ = SI_1mV_ ~70% of MSO.
